# Alanine, a potential amino acid biomarker of pediatric sepsis: a pilot study in PICU

**DOI:** 10.1007/s00726-024-03408-3

**Published:** 2024-07-27

**Authors:** Tiantian Liu, Yaya Xu, Shaohua Hu, Shuyun Feng, Hong Zhang, Xiaodong Zhu, Chunxia Wang

**Affiliations:** 1 Department of Critical Care Medicine, Shanghai Children’s Hospital, Shanghai Jiao Tong University School of Medicine, No. 355 Luding Road, Putuo District, Shanghai, 200062 China; 2 Institute of Pediatric Infection, Immunity, and Critical Care Medicine, Shanghai Children’s Hospital, Shanghai Jiao Tong University School of Medicine, Shanghai, 200062 China; 3 Department of Pediatric Critical Care Medicine, Xinhua Hospital, Shanghai Jiao Tong University School of Medicine, Shanghai, 200092 China; 4 Department of Clinical Laboratory, Shanghai Children’s Hospital, Shanghai Jiao Tong University School of Medicine, Shanghai, 200062 China; 5 Clinical Research Unit, Shanghai Children’s Hospital, Shanghai Jiao Tong University School of Medicine, Shanghai, 200062 China

**Keywords:** Amino acid profile, Alanine, Diagnostic biomarker, Sepsis, Children

## Abstract

Sepsis is characterized by a metabolic disorder of amino acid occurs in the early stage; however, the profile of serum amino acids and their alterations associated with the onset of sepsis remain unclear. Thus, our objective is to identify the specific kinds of amino acids as diagnostic biomarkers in pediatric patients with sepsis. Serum samples were collected from patients with sepsis admitted to the pediatric intensive care unit (PICU) between January 2019 and December 2019 on the 1^st^, 3^rd^ and 7^th^ day following admission. Demographic and laboratory variables were also retrieved from the medical records specified times. Serum amino acid concentrations were detected by UPLC-MS/MS system. PLS-DA (VIP > 1.0) and *Kruskal-Wallis* test (*p* < 0.05) were employed to identify potential biomarkers. *Spearman’s* rank correlation analysis was conducted to find the potential association between amino acid levels and clinical features. The diagnostic utility for pediatric sepsis was assessed using receiver operating characteristic (*ROC*) curve analysis. Most of amino acid contents in serum were significantly decreased in patients with sepsis, but approached normal levels by the seventh day post-diagnosis. Threonine (THR), lysine (LYS), valine (VAL) and alanine (ALA) emerged as potential biomarkers related for sepsis occurrence, though they were not associated with PELOD/PELOD-2 scores. Moreover, alterations in serum THR, LYS and ALA were linked to complications of brain injury, and serum ALA levels were also related to sepsis-associated acute kidney injury. Further analysis revealed that ALA was significantly correlated with the *Glasgow* score, serum lactate and glucose levels, C-reactive protein (CRP), and other indicators for liver or kidney dysfunction. Notably, the area under the *ROC* curve (AUC) for ALA in distinguishing sepsis from healthy controls was 0.977 (95% *CI*: 0.925-1.000). The serum amino acid profile of children with sepsis is significantly altered compared to that of healthy controls. Notably, ALA shows promise as a potential biomarker for the early diagnosis in septic children.

**Supplementary Information:**

The online version contains supplementary material available at 10.1007/s00726-024-03408-3.

## Introduction

Sepsis is commonly encountered and often fatal among children all over the world (Fleischmann-Struzek et al. 2018). In China, the estimated incidence was 118 cases per 100 000 person-years (Wang et al. 2014). The most recent definition of sepsis describes it as life-threatening organ dysfunction resulting from a dysregulated host response to infection (Singer et al. 2016). Approximately three decades ago, it was recognized that disturbances in plasma amino acid profiles were indicative of the host response during sepsis (Vente et al. 1989). To date, research on amino acids in relation to sepsis remains limited in either adults or children (Su et al. 2015; Spanaki et al. 2018; Reisinger et al. 2021; Chen et al. 2022). It is uncertain whether the changes in amino acid profiles are specific, and which amino acids are associated with severity or outcomes in patients with sepsis. Considering the important roles of amino acids in defending against pathogens and mitigating the hyperinflammation (Spanaki et al. 2018; Tomé 2021), exploring the potential clinical value of blood amino acids could provide new insights into the early diagnosis of sepsis, the assessment of its severity or the effectiveness of treatments.

Recent preliminary findings in adults with sepsis suggest that the alterations in amino acid profiles, when combined with other biomarkers, may contribute to the early diagnosis of sepsis (Mierzchala-Pasierb et al. 2020). Furthermore, a multivariate index comprising the kynurenine / tryptophan (KYN/TRP) ratio, arginine (ARG) and phenylalanine (PHE) could differentiate sepsis from systemic inflammatory response syndrome (SIRS) or healthy controls (Ahn et al. 2021). Additionally, the metabolism of PHE, tyrosine (TYR), and TRP biosynthesis are distinct in septic patients compared with healthy controls (Chen et al. 2022). Besides of adults, decreased levels of ARG, citrulline (CIT), ornithine (ORN) and the ARG / ORN have been observed in septic children (Weiss et al. 2012). Moreover, plasma CIT levels predict the risk of bacteremia in children with acute lymphoblastic leukemia (Pietri et al. 2021). Thus, we hypothesize that amino acids might serve as potential biomarker for predicting the occurrence or severity of sepsis. However, serum dynamic amino acid profiles in septic children are still largely unclear until now.

In this study, twenty amino acids (proteinogenic amino acids) were specifically detected as targets in serum of septic children and healthy controls by an ultraperformance liquid chromatography coupled to tandem mass spectrometry (UPLC-MS/MS) system. Here, we aimed to identify the candidate amino acids as novel biomarkers for the early diagnosis of pediatric sepsis.

## Methods

### Patients

Patients with sepsis admitted to the pediatric intensive care unit (PICU) between January 2019 and December 2019 were eligible for the study. The definition of pediatric sepsis followed the criteria established by the International Pediatric Sepsis Consensus Conference in 2005 (Goldstein et al. 2005). The inclusion criteria included (1) aged with 1 month to 18 years old, (2) diagnosis of sepsis within 24 h of admission to the PICU, (3) PICU stay exceeding 7 days. The exclusion criteria included (1) advanced tumors, (2) hereditary metabolic diseases. Septic blood samples were collected at three points: within 24 h of diagnosis (S0), on the 3^rd^ day (S3) and on the 7^th^ day (S7). Twelve residual serum samples from healthy children undergone routine physical examinations were used as control with normal laboratory finding or without underlying disease. The healthy blood samples were collected after fasting at night for at least 10 h. Sepsis-associated organ dysfunction including brain injury, acute kidney injury (AKI) and liver dysfunction criteria were relied on International Pediatric Sepsis Consensus Conference in 2005 (Goldstein et al. 2005). The study protocol was approved by the Ethics Committee of Xinhua Hospital affiliated to Shanghai Jiao Tong University School of Medicine (approval number: XHEC-C-2019-060). It was conducted in compliance with local regulatory requirements, Good Clinical Practice (GCP), and the Declaration of Helsinki (Association 2013). The informed consent was signed by the patients’ parents or relatives.

### Variables

Demographic data, clinical features, laboratory variables, and outcomes were collected at the time points of blood sampling including S0, S3 and S7. The clinical features included respiratory rate, heart rate, mean arterial pressure (MAP), systolic blood pressure (SBP), complications, primary infection site, *Glasgow* score, and outcomes in septic pediatrics. Laboratory indexes were collected including inflammatory response-related factors (C-reactive protein [CRP], procalcitonin [PCT], and white blood cell [WBC]), indicators for liver function (total bilirubin [TBIL], alanine aminotransaminase [ALT], aspartate aminotransferase [AST], γ-glutamyltransferase [γ-GT], blood ammonia and albumin [ALB]), indicators for coagulation function (prothrombin time [PT], international normalized ratio [INR], and fibrinogen [Fib]), indicators for kidney function (blood urea nitrogen [BUN] and creatinine [Cr], and arterial blood gas (lactate and glucose) data.

### Serum amino acids spectrum detection

Total amino acids were extracted from serum using amino acid extraction reagent and transformation solution was derivatized. LC/MS/MS (Shimadzu LCMS-8040CL) was used for the determination by professional inspectors at the laboratory of Shanghai Children’s Hospital. Reagents, standard products and quality control products were sourced from Guangzhou CLINMETA Medical Device Co., LTD.

### Sample preparation and LC/MS analysis

On the Biomek 4000 workstation (Biomek 4000, Beckman Coulter, Inc., Brea, California, USA), a mixture involving 20 µL of serum / quality control products and 80 µL of dilution reagents was first centrifuged. Subsequently, 100 µL of internal standards and 10 µL of diluents were vigorously vortexed for 5 min. Following centrifugation and nitrogen blow drying at 50 ℃ and 40LPM for 10 min, 50 µL of the supernatant was treated with 500 µL of freshly prepared derivatizing agents. Post-derivatization and another nitrogen blow drying phase at 60 ℃ and 40LPM for 30 min, 100 µL of an amino acid complex solution was added, sealed and agitated at room temperature for 5 min. Subsequently, 2 µL derivatized samples and serial dilutions of derivatized stock standards were randomly analyzed and quantitated by UPLC-MS/MS system (ACQUITY UPLC-Xevo TQ-S, WatersCorp., Milford, MA, USA) to quantitate all targeted metabolites. Standards of targeted metabolites were sourced from Sigma-Aldrich (St. Louis, MO, USA), Steraloids Inc. (Newport, RI, USA) and TRC Chemicals (Toronto, ON, Canada).

### Statistical analysis

For data processing, the raw data files generated by UPLC-MS/MS were processed using MassLynx software (v4.1, Waters, Milford, MA, USA) to conduct peak integration, calibration, and quantitation of each metabolite. Partial least squares discriminant analysis (PLS-DA) including score plot, loading plot and variable importance in projection (VIP) values, along with the heatmap and correlation analysis, was performed by MetaboAnalyst 5.0 using the online server. Laboratory variables and serum amino acid levels were presented as median (interquartile rang, IQR). Correlation analyses were assessed by *Spearman’s* rank correlation. Correlation heatmap and receiver operating characteristic (*ROC*) curve were generated using the Xiantao tool (https://www.xiantao.love/). Data analysis was carried out with using GraphPad Prism version 8.0 (GraphPad Software, San Diego, CA), R software (version 4.0.2) and SPSS 26.0 (IBM Corp., Armonk, NY). Significant differences were analyzed by student’s *t* test or *Kruskal-Wallis* test. Values of *p* < 0.05 were considered statistically significant.

## Results

### Baseline characteristics

The clinical baseline characteristics are presented as Table 1. The study included 12 healthy normal children and 25 septic children. The median age of children with sepsis was 45 (15–84) months. There were no significant differences in terms of age, gender, or body weight between the groups. Notably, 96% of the septic children had complications associated with liver injury. The median of *Glasgow* score was 15 (range: 10.75-15). Out of the septic children, 23 survived, resulting in a survival rate of 92% (23/25). Additionally, 36% of the cases received mechanical ventilator or continuous renal replacement therapy (CRRT) support, and 40% needed vasoactive agents (Table 1).

**Table 1:** Baseline characteristics in children with sepsis in this study for determining the serum amino acid levels

Characteristics	Normal (*n* = 12)	Sepsis (*n* = 25)	*P* value
**Age**,** month**	51 (17–93)	45 (15–84)	0.663
**Male**,** n (%)**	6 (50)	17 (68)	0.581
**Body weight**,** kg**	17 (9–26)	14 (10–22)	0.343
**Respiratory rate**,** /min**		33 (30–36)	
**Heart rate**,** beats/min**		138 (120–154)	
**MAP**,** mmHg**		60 (55.5–67)	
**SBP**,** mmHg**		100 (87.5–107)	
***Glasgow *** **score**		15 (10.75-15)	
**PELOD**		2.0 (1.0-10.75)	
**PELOD-2**		4.0 (2.0–5.0)	
**Complications**			
Respiratory failure, n (%)		10 (40)	
Brain injury, n (%)		5 (25)	
Liver injury, n (%)		24 (96)	
Kidney injury, n (%)		4 (16)	
Hematologic dysfunction, n (%)		1 (4)	
**Primary infection site**			
Respiratory tract, n (%)		4 (16)	
Lung, n (%)		14 (56)	
Digestive tract, n (%)		5 (20)	
Central nervous system, n (%)		3 (12)	
**Mechanical ventilator**,** n (%)**		9 (36)	
**CRRT**,** n (%)**		9 (36)	
**Vasoactive agents**,** n (%)**		12 (48)	
**PICU survivor**,** n (%)**		23 (92)	

*Abbreviations* MAP: Mean arterial pressure; SBP: Systolic blood pressure; P-MODS: Pediatric multiple organ dysfunction syndrome; PICU: Pediatric intensive care unit; CRRT: Continuous renal replacement therapy. Data are presented as median (interquartile range, IQR)

### Serum amino acid profiles

Upon admission to PICU (S0), most amino acid levels were significantly lower compared to those in healthy controls. By the third day (S3), the concentrations of lysine (LYS) and threonine (THR) had increased, while by the seventh day (S7), levels of glycine (GLY), LYS, glutamic acid (GLU), proline (PRO), TYR, aspartic acid (ASP), CIT and THR were increased relative to S0. Conversely, the concentrations of alanine (ALA), valine (VAL), serine (SER), leucine (LEU), ARG, TRP, histidine (HIS) and aspartic acid (ASP) remained lower than the healthy normals at all three tested points. Phenylalanine (PHE) level was decreased at S7 compared with the healthy normal. There were no significant differences in serum ORN levels between septic patients and normal control group (Table 2).

**Table 2:** Serum amino acid concentrations in the normal and sepsis groups

Amino acid	Normal (*n* = 12)	Sepsis (*n* = 25)			*P* value
		S0	S3	S7	
Glycine, µmol/L	300.4 (275.0-353.4)	188.7 (117.4-222.3) ^*^	242.5 (175.6-313.7)	253.6 (200.7-288.1) ^*^	< 0.001
Lysine, µmol/L	225.4 (194.0-234.9)	117.0 (90.10-163.9) ^*^	193.4 (135.5-319.6) ^#^	235.8 (145.2-318.1) ^#^	< 0.001
Glutamic acid, µmol/L	217.0 (203.0-224.4)	178.3 (155.2-207.6) ^*^	193.2 (174.8-206.5)	213.4 (181.0-257.0) ^#^	0.003
Proline, µmol/L	194.6 (144.0-286.6)	95.49 (76.28–123.3) ^*^	158.8 (98.31–187.4)	169.7 (94.12–200.3) ^#^	< 0.001
Tyrosine, µmol/L	83.31 (65.56–99.23)	47.36 (39.49–58.58) ^*^	57.86 (46.82–75.43)	55.03 (45.29–67.29) ^#^	< 0.001
Asparagine, µmol/L	47.85 (41.89–56.22)	20.65 (15.95–32.60) ^*^	33.53 (21.12–43.04)	35.67 (24.60-52.86) ^#^	< 0.001
Citrulline, µmol/L	27.80 (25.05–31.14)	8.535 (5.979–13.06) ^*^	10.72 (8.786–13.17) ^*^	13.29 (9.698–19.33) ^* #^	< 0.001
Threonine, µmol/L	121.0 (95.24–149.9)	46.78 (36.33–98.17)	147.8 (56.84–208.3) ^#^	179.5 (69.62–275.6) ^#^	< 0.001
Alanine, µmol/L	412.5 (359.6-477.5)	170.2 (102.2-207.4) ^*^	190.0 (124.2–231.0) ^*^	181.8 (151.0-290.1) ^*^	< 0.001
Valine, µmol/L	308.0 (254.9-369.4)	192.7 (144.1-255.4) ^*^	221.1 (162.0-294.7) ^*^	208.3 (179.2-297.2) ^*^	0.003
Serine, µmol/L	198.4 (175.3-219.3)	105.4 (69.81–120.4) ^*^	130.9 (91.94–159.5) ^*^	138.4 (90.75–167.6) ^*^	< 0.001
Leucine, µmol/L	166.1 (132.2-187.3)	89.93 (75.74–147.9) ^*^	122.2 (91.70-157.1)	103.8 (92.65–138.7) ^*^	< 0.001
Arginine, µmol/L	128.7 (113.6-143.8)	24.64 (11.67–39.84) ^*^	47.60 (32.05–83.83) ^*^	53.88 (24.30-96.71) ^*^	< 0.001
Tryptophan, µmol/L	99.95 (70.49–118.8)	27.35 (15.26–51.08) ^*^	54.40 (21.95–72.55) ^*^	45.62 (26.10–77.40) ^*^	0.001
Histidine, µmol/L	94.47 (81.98–101.0)	55.87 (45.63–73.03) ^*^	61.68 (49,64-69.62) ^*^	60.35 (53.46–70.53) ^*^	< 0.001
Aspartic acid, µmol/L	38.92 (31.47–41.90)	14.44 (11.22–21.86) ^*^	15.45 (12.07–20.71) ^*^	15.60 (10.98–23.45) ^*^	< 0.001
Isoleucine, µmol/L	71.22 (63.86–94.51)	40.42 (27.39–70.39) ^*^	47.71 (39.02–66.90)	50.42 (39.40-65.38)	0.009
Methionine, µmol/L	30.92 (22.41–38.01)	12.66 (9.051–19.57) ^*^	23.14 (14.07–30.33)	18.70 (11.52–30.22)	0.001
Phenylalanine, µmol/L	103.5 (92.01–128.0)	92.61 (72.27–119.1)	87.49 (67.51–108.3)	76.83 (59.61–88.50) ^*^	0.005
Ornithine, µmol/L	71.49 (56.20-87.75)	51.65 (40.82–82.88)	68.71 (44.47–107.2)	84.17 (51.59–103.7)	0.145

Data are expressed as median (IQR) and were analyzed by *Kruskal-Wallis* test from data of four independent groups including normal controls, within 24 h of diagnosis (S0), on the 3^rd^ day (S3), and on the 7^th^ day (S7). ^*^ indicates the significant difference compared with Normal Control group, # indicates the significant difference compared with S0 group.

### Selected amino acids as potential biomarkers for sepsis

**Fig. 1:**
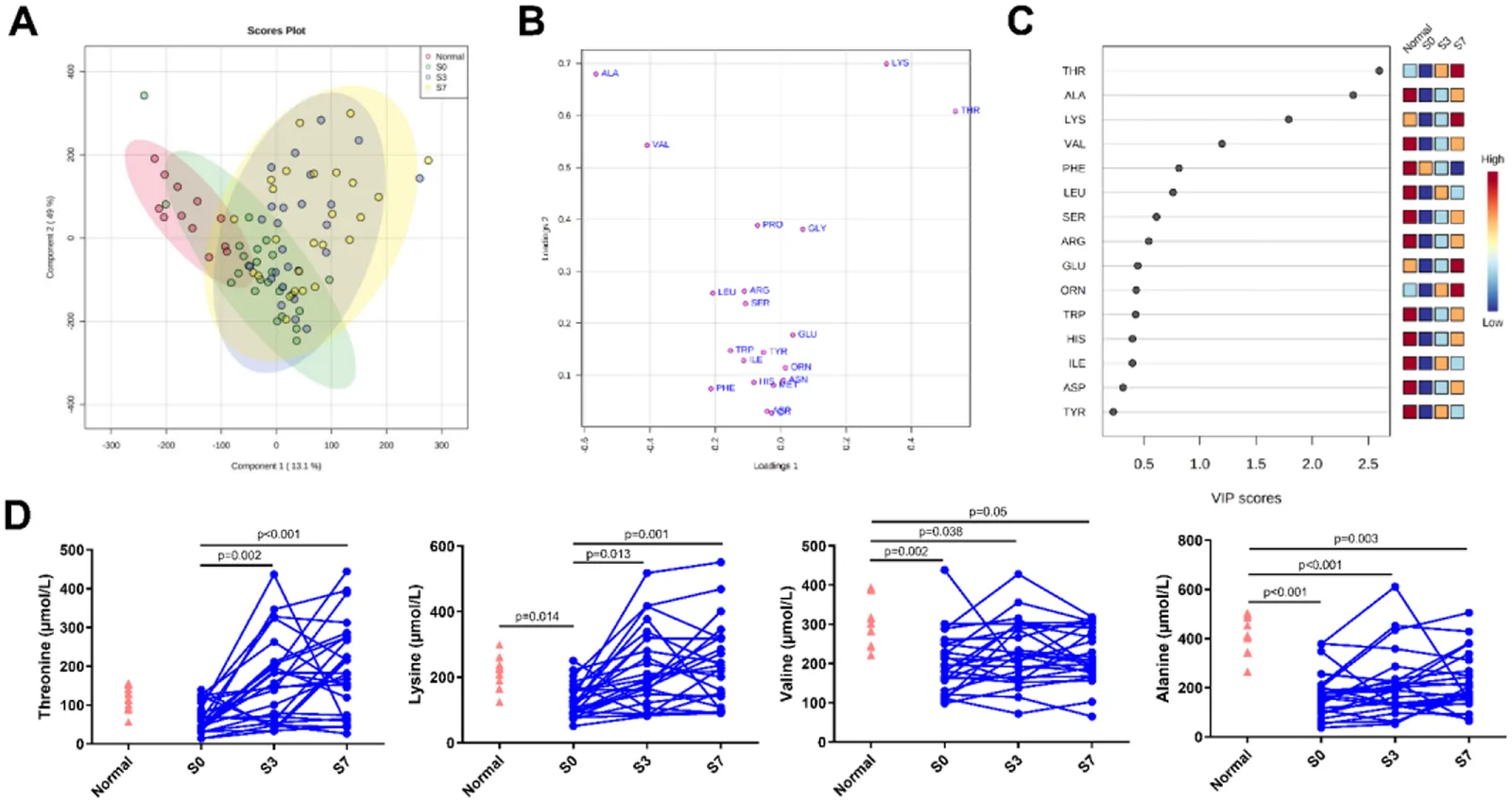
Serum amino acid profiles in healthy controls and patients with sepsis. (**A**). Scores plot in Partial least squares discriminant analysis (PLS-DA), (**B**). Loading plot in PLS-DA, (**C**). VIP scores plot in PLS-DA, (**D**). Line graph illustrates altered concentrations of serum threonine (THR), lysine (LYS), valine (VAL), and alanine (ALA) in healthy controls and patients with sepsis at the indicated time point (S0, S3, and S7). S0, serum collected within 24 h of enrollment, S3, 3^rd^ day of enrollment and S7, 7^th^ day of enrollment in PICU

The PLS-DA score plot indicated that the normal group was clearly distinguishable from S0, S3, and S7 groups, respectively (Fig. 1A). The loading plot revealed significant variations in the levels of THR, LYS, VAL and ALA among patients with sepsis compared to the normal group (Fig. 1B), with the VIP values of these four metabolites exceeding 1.0, as shown in the importance plot (Fig. 1C). In addition, the concentrations of LYS, ALA, THR and VAL were statistically different between septic patients and healthy controls (all *p* < 0.05, Table 2). Moreover, the concentrations of THR, ALA, LYS, and VAL were significantly reverted to near-normal levels from S0 to S7 (Fig. 1D).

### Selected amino acids are associated with sepsis-associated brain or kidney injury

**Fig. 2:**
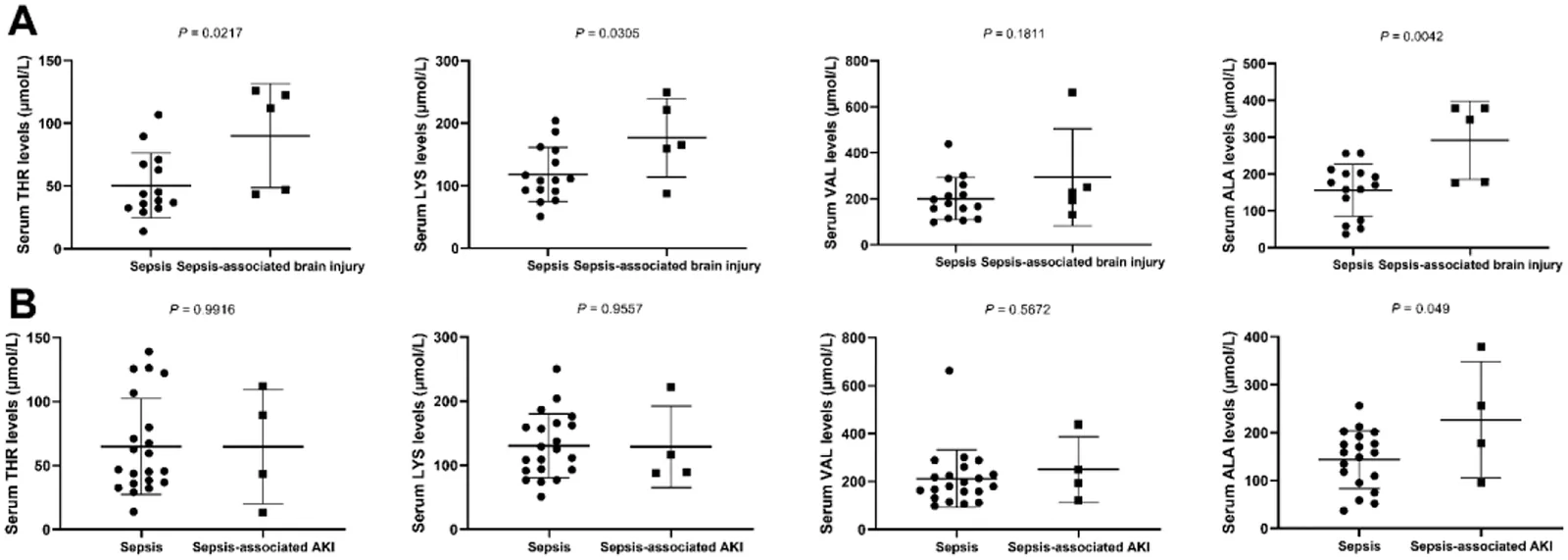
Comparison of serum THR, LYS, VAL and ALA levels in septic patients. (**A**). sepsis vs. sepsis-associated brain injury, (**B**). sepsis vs. sepsis-associated AKI. Abbreviations: THR: threonine; LYS: lysine; VAL: valine; ALA: alanine; AKI: acute kidney injury. Serum was collected within 24 h of enrollment (S0). Data was analyzed by Student’s *t* test, *p* < 0.05 indicated significantly difference

The laboratory indices revealed significant recovery in the levels of CRP (*p* < 0.001), PCT (*p* = 0.005), PT (*p* = 0.014), Fib (*p* < 0.001), and ALB (*p* = 0.037) were significantly recovered from S0 to S3 and S7 in patients with sepsis. No significant changes were observed in liver and kidney function indictors, arterial blood gas data, WBC, and INR (Table 3). Furthermore, patients with sepsis-associated brain injury exhibited higher levels of THR, LYS, and ALA compared to those in patients without brain injury (Fig. 2A), with ALA levels also being elevated in patients with sepsis-associated AKI compared to those without AKI at S0 (Fig. 2B). However, there was no correlation between the concentrations of selected amino acids (THR, LYS, VAL and ALA) concentrations and the PELOD or the PELOD-2 scores (Supplementary Fig. 1).

**Table 3:** The changes of laboratory indexes in children during sepsis

Parameters	S0 (*n* = 25)	S3 (*n* = 25)	S7 (*n* = 25)	*P* value
**Indicators for inflammatory response**				
CRP, mg/L	107 (27–161)	78 (22–159)	16 (7-42.5)	< 0.001
PCT, ng/mL	10.44 (0.62–24.71)	2.25 (0.54–10.99)	0.4 (0.24–1.025)	0.005
WBC, ×10^9^/L	8.78 (3.99–16.51)	7.8 (3.64–12.96)	8.5 (5.2-12.76)	0.803
**Indicators for liver function**				
TBIL, µmol/L	12.2 (8.35-16)	8 (4.55–15.38)	9.8 (5.6–16.1)	0.268
ALT, U/L	34 (24–107)	43 (22–77)	37 (24–71)	0.969
AST, U/L	56 (22.5-99.25)	60 (24.75–102.5)	65 (38–112)	0.91
γ-GT, U/L	63 (18–160)	39 (14.75–114.5)	38 (21.5-80.25)	0.781
Blood ammonia, µmol/L	26 (14.25-41.0)	22.5 (11-42.25)	21.5 (15.5–38.5)	0.842
ALB, g/dL	34.5 (31.15–40.75)	32.6 (29.2–35.3)	35.1 (33.0-38.3)	0.037
**Indicators for coagulation function**				
PT, s	15 (12.2-15.85)	12.2 (11.05–14.7)	12.7 (11.7–15.1)	0.014
INR	1.37 (1.125–1.445)	1.26 (1.05–1.49)	1.15 (1.08–1.25)	0.114
Fib, g/L	3.17 (2.52–5.42)	2.33 (1.95–2.94)	1.92 (1.69–2.29)	< 0.001
**Indicators for kidney function**				
BUN, mmol/L	5.13 (3–11)	3.95 (2-5.75)	4 (2–6)	0.12
Cr, µmol/L	33.9 (26.1-53.55)	24.8 (19.4-37.05)	22 (19.2–37.1)	0.052
**Arterial blood gases**				
Lactate, mmol/L	1.4 (0.7–1.9)	1.0 (0.7–1.15)	0.8 (0.7–1.45)	0.364
Glucose, mg/dL	6.1 (5.6–7.3)	6.1 (5.4–8.2)	6.1 (5.85–6.9)	0.995

*Abbreviations* CRP: C-reactive protein; PCT: Procalcitonin; WBC: White blood cell; TBIL: Total bilirubin; ALT: Alanine aminotransaminase; AST: Aspartate aminotransferase; γ-GT: γ-Glutamyltransferase; ALB: Albumin; PT: Prothrombin time; INR: International normalized ratio; Fib: Fibrinogen; BUN: Blood urea nitrogen; Cr: Creatinine. Data are expressed as median (IQR) and were analyzed by *Kruskal*-*Wallis* test from data of three independent groups including within 24 h of diagnosis (S0), on the 3^rd^ day (S3), and on the 7^th^ day (S7)

### Correlation analysis of serum amino acids with inflammatory response indicators and organ function in septic patients

**Fig. 3:**
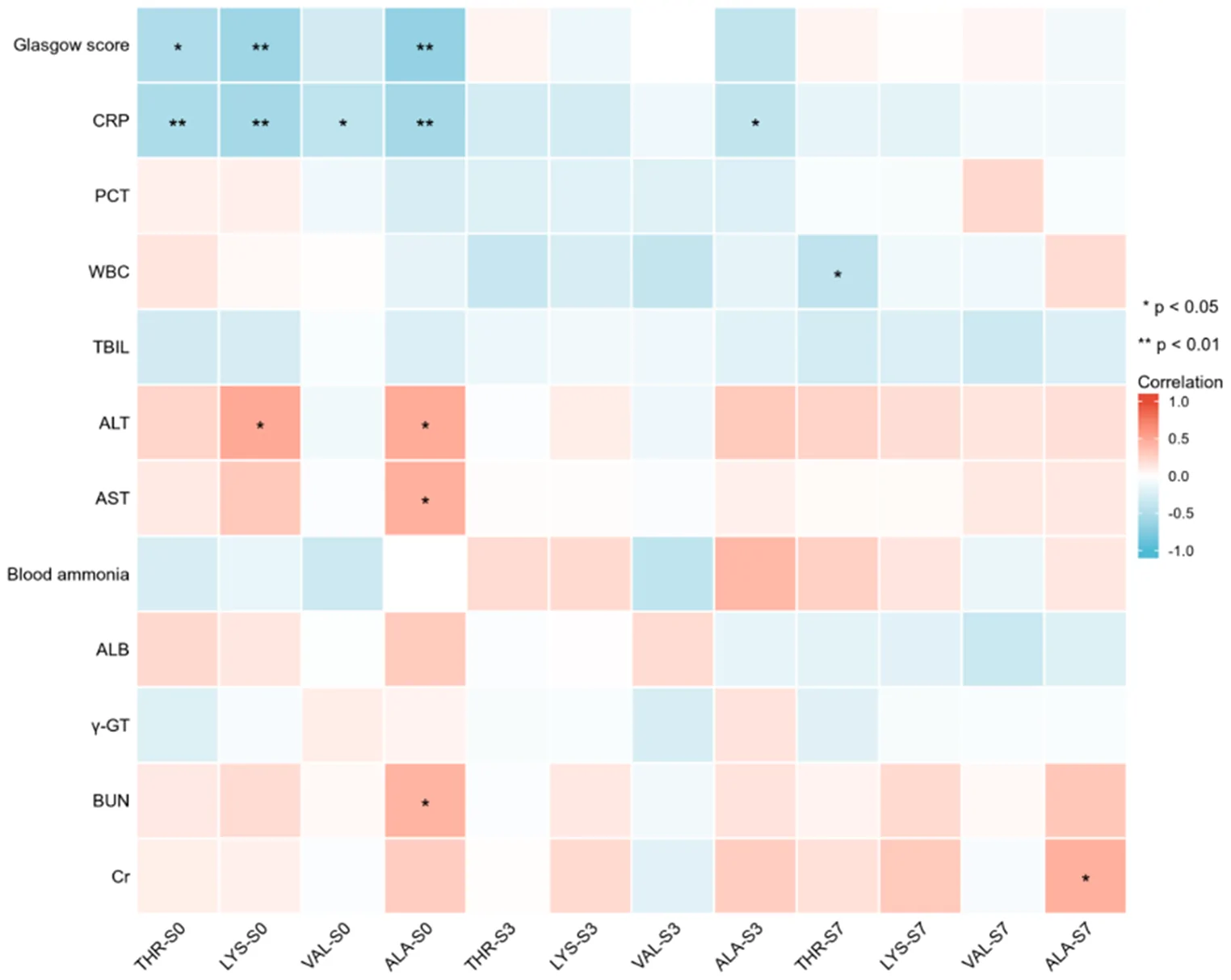
Correlation heatmap analysis of serum amino acid levels with the clinical indexes in septic patients. The red color indicated positive correlation and the blue color indicated negative correlation. The depth of color represents the magnitude of *r*. ^*^ indicated *p* < 0.05, ^**^ indicated *p* < 0.01. S0, serum collected within 24 h of enrollment, S3, 3^rd^ day of enrollment and S7, 7^th^ day of enrollment in PICU

**Fig. 4:**
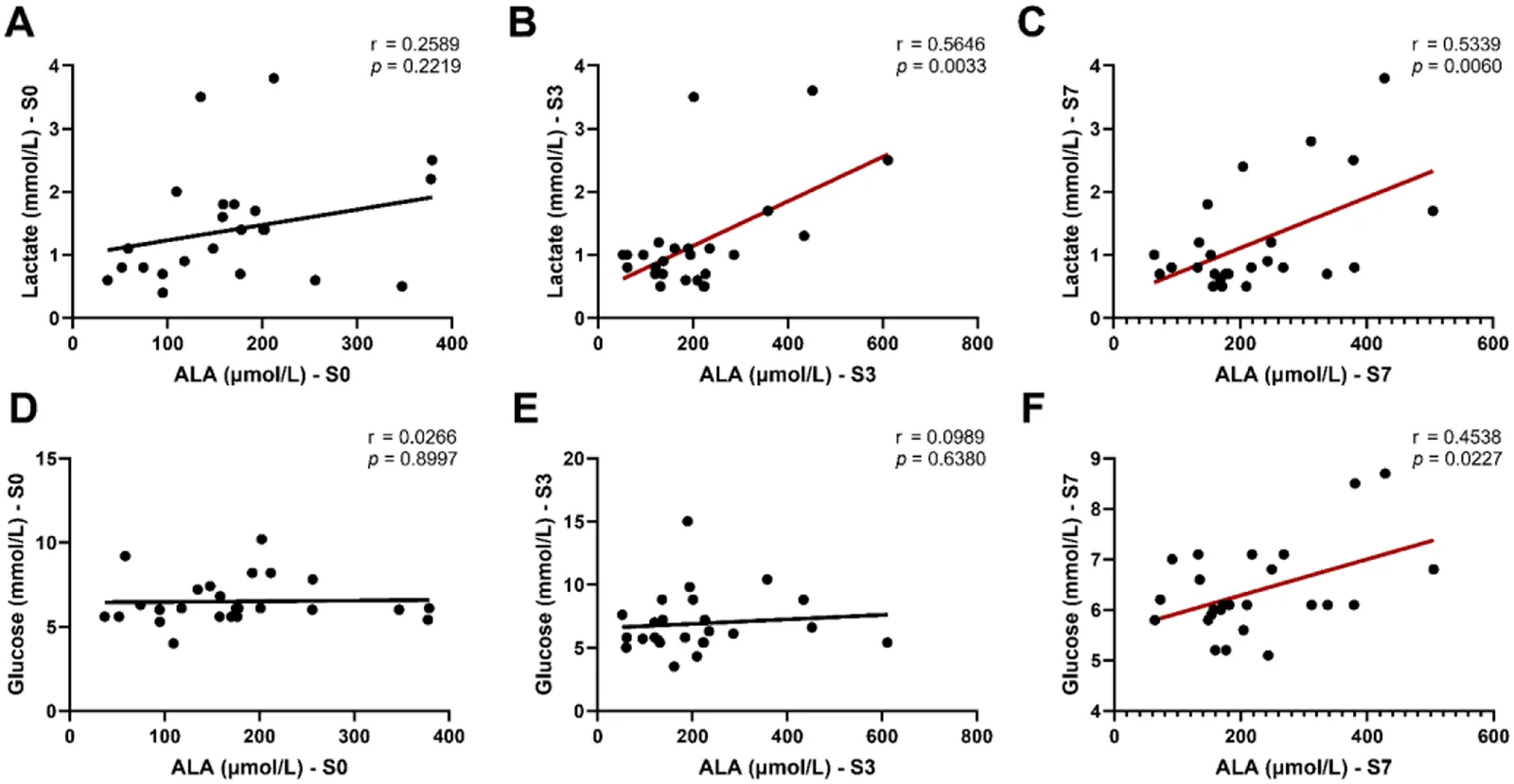
*Spearman*’s rank correlation analysis of serum ALA with arterial blood lactate and glucose. (**A**, **D**) Data were collected within 24 h of enrollment (S0); (**B**, **E**) Data were collected at 3^rd^ day of enrollment (S3); (**C**, **F**) Data were collected at 7^th^ day of enrollment in PICU (S7)

The correlation heatmap analysis showed that THR, LYS and ALA were negatively correlated with the *Glasgow* score. All these 4 kinds of amino acids were negatively correlated with CRP at S0, but only ALA maintained this negative correlation with CRP at S3. Only THR was negatively correlated with WBC at S7. Nevertheless, the relationship between PCT and these 4 kinds of amino acids were not significant. Moreover, LYS was positively correlated with ALT, and ALA was positively correlated with ALT, AST, BUN, and ALB at S0, and positively correlated with Cr at S7 (Fig. 3). Considering ALA’s role as a glucose precursor, serum ALA levels were positively associated with arterial blood lactate at S3 and S7, and with arterial blood glucose at S7 (Fig. 4).

### Serum ALA as an effective diagnostic biomarker for pediatric sepsis

**Fig. 5:**
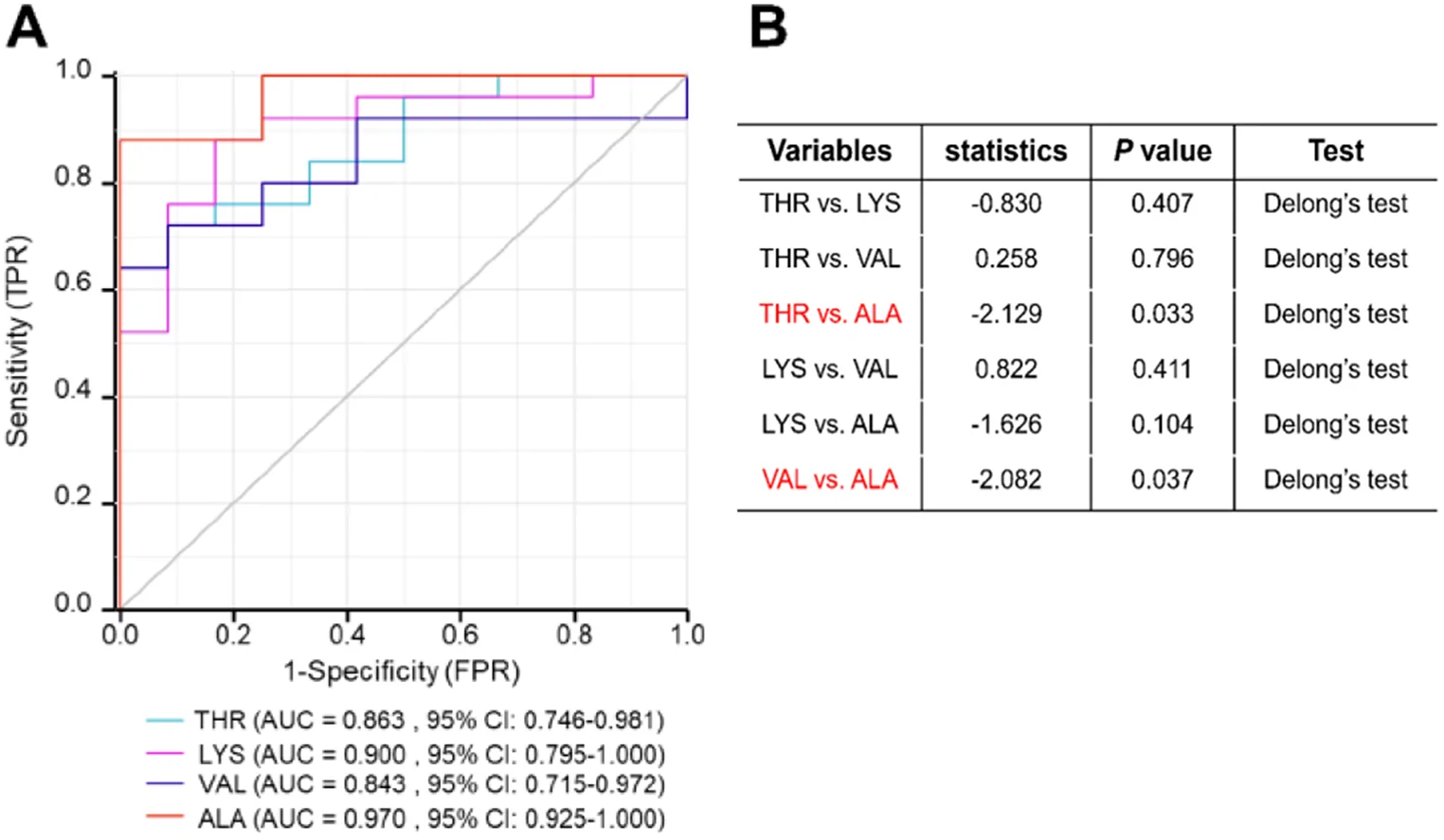
The AUC of THR, LYS, VAL and ALA for discriminating patients with sepsis from healthy control. (**A**). *ROC* curve at S0. (**B**). Statistical comparison between different kind of amino acids. Abbreviations: THR: threonine; LYS: lysine; VAL: valine; ALA: alanine; AUC: area under the receiving operating characteristic curve; *CI*: confidence interval

The area under the receiver operating characteristic curve (*AUC*) for THR, LYS, VAL and ALA in distinguishing sepsis from healthy control was 0.863 (95% *CI*: 0.746–0.981), 0.9 (95% *CI*: 0.795-1.000), 0.843 (95% *CI*: 0.715–0.972) and 0.97 (95% *CI*: 0.925-1.000), respectively (Fig. 5A). The diagnostic efficacy of ALA for sepsis was superior to both THR and VAL (Fig. 5B). Furthermore, ALA was identified as the most effective serum amino acid for diagnosing sepsis (Table 4).

**Table 4:** Areas under the *ROC* curves for serum THR, LYS, VAL, and ALA as diagnostic biomarkers for sepsis

Variables	AUC	95% *CI*	Cut-off	Sensitivity	Specificity
THR	0.863	0.746–0.981	84.250	0.720	0.947
LYS	0.900	0.795-1.000	188.041	0.880	0.833
VAL	0.843	0.715–0.972	219.846	0.640	1.000
ALA	0.970	0.925-1.000	261.077	0.880	1.000

*Abbreviations* THR: Threonine; LYS: lysine; VAL: Valine; ALA: Alanine; AUC: Area under the receiving operating characteristic curve; *CI*: Confidence interval

## Discussion

In early stage of sepsis, increased hepatic amino acid uptake is beneficial for hepatic gluconeogenesis, which is important for hepatic energy and oxygen demand. When sepsis-associated liver dysfunction occurred, the capacity of hepatic amino acid uptake decreased (Träger et al. 2003). Additionally, hyperglycemia, high protein and fat catabolism, highly amplified protein synthesis, and increased oxygen uptake and demands are associated with sepsis occurrences (Pravda 2014; Plummer and Deane 2016; Chioléro et al. 1997). Among metabolic disorder, amino acid disorder occurs in the early stage of sepsis. In our study, except of PHE and ORN, almost all kinds of amino acids were generally decreased in serum. We suspected that the general depletion of amino acids could be one of characteristics during sepsis, resulting from the combined effects of host liver or immune cells responses and infection-induced disturbed amino acid metabolism (Sax et al. 1988; Biolo et al. 1997; Sun et al. 2023). Among them, ALA is expected to be an effective biomarker for the early diagnosis of pediatric sepsis. To the best of our knowledge, it is the first report about exploring serum amino acid profile to reveal the potential value of serum ALA in septic children.

In terms of methodology, PLS-DA (VIP > 1.0) and *Kruskal-Wallis* test (*p* < 0.05) were employed to select candidate amino acids from amino acid spectrum data. Only one non-essential amino acid (ALA) and three essential amino acids (THR, LYS and VAL) were selected. These four amino acids were negatively correlated with CRP, implying an association with the inflammatory response. In septic rat models, the utilization of THR is increased for synthesizing acute phase proteins, intestinal proteins, and mucins (Faure et al. 2007), positioning THR as a potential biomarker for Lassa fever and Ebola (Gale et al. 2020). LYS is linked to the production of nitric oxide production (Liaudet et al. 1997) and oxidative stress injury in sepsis (Zhang et al. 2019). A recent study shows that branched chain amino acids (BCAAs) including LEU, ILE and VAL are associated with the development and persistence of cardiovascular organ failure in septic shock (Puskarich et al. 2021). ALA possesses a gluconeogenic function, which is associated with bacterial killing ability (Peng et al. 2015). Thus, THR, LYS, VAL and ALA may be closely related to the pathological mechanisms of sepsis, particularly infection stress.

In our study, the level of ALA was significantly lower in septic patients than the normal controls, which might be associated increased levels of inflammatory cytokine, higher catecholamines and immune response in the early stage of sepsis, and ALA is vital in gluconeogenesis in the liver (Holeček 2024). One of outstanding characteristic of patients undergoing total hip replacement is hypoalaninaemia and increased removal of intravenous ALA (Elia et al. 1980). Among 4 kinds of amino acids, the AUC of ALA for discriminating sepsis was 0.977 (0.925-1.000), demonstrating optimal capacity for the early diagnosis of sepsis. When the cut-off value was 261.077 µmol/L, the sensitivity was 0.880 and the specificity is 1.000. The result is significantly better than that of PCT, and combination with other biomarkers with high specificity for sepsis can be considered as an effective and feasible diagnostic method. However, whether ALA could be an effective biomarker for infection stress in sepsis needs a prospective study design in a larger population.

Plasma or serum amino acid alterations are closely associated with organ injury and inflammatory responses. Hepatic, brain and renal functions depend on nitrogen balance. ALA was found to positively correlate with ALT, AST, BUN and Cr levels, negatively correlate with the *Glasgow* score, and was elevated in cases of sepsis-associated with brain injury and AKI. One potential explanation for ALA’s correlation with sepsis-induced elevations in ALT and AST could be ALA’s primary synthesis in the liver (Hou et al. 2020). In the brain and kidney, ALA is metabolized to produce ammonia, and formed by transamination then added to venous blood (Pitts and Stone 1967; Bröer et al. 2007) and ALT promoting ALA increase was related to cellular oxygen availability (Laustsen et al. 2014). ALA and glutamine induced ammonia imbalance could impair brain function (Coqueiro et al. 2018; Dadsetan et al. 2013). It is important that L-alanine-L-glutamine infusion dose was determined to increase brain glutamine and ALA concentrations in patients with severe traumatic brain injury (Nägeli et al. 2014). In the alloxan induced diabetic rats, ALA supplementation reversed kidney damage possibly due to enhanced glomerular filtration rate or urea cycle modulation, and ALA promoted the synthesis of GSH and decrease the damage of reactive oxygen species (Dandare et al. 2021). In addition, ALA production is associated with renal fibrosis (Nielsen et al. 2020). Although the serum levels of ALA were increased in septic patients with brain injury or AKI. However, its value was still far below the values of the healthy controls, indicating the clinical ALA usability is limited. Dynamic monitoring the changes of serum ALA levels during sepsis or combination of serum ALA levels with other clinical indicators might be better for early identifying and preventing sepsis with brain injury or AKI.

Besides, glucose and ALA are the main sources of lactate in the body (Adeva-Andany et al. 2014). At early (8 h) and late (18 h) sepsis in rats with cecal ligation and puncture, gluconeogenesis from ALA, lactate and pyruvate was decreased (Souza Galia et al. 2021). However, in our study, serum ALA level was positively related to arterial blood lactate on the 3^rd^ and 7^th^ day following admission, and glucose only on the 7^th^ day following admission, indicating low ALA is not caused by gluconeogenesis. The decrease of ALA in sepsis might be ALA hydrolysis caused by enzyme of bacteria or virus and participating in host immune response (Peng et al. 2024) or increased hepatic uptake ALA in sepsis, but the hypothesis needs to be tested experimentally. Thus, further studies are needed to elucidate the molecular mechanism and clinical value between ALA and liver, brain and kidney injury and glucose and lactate metabolism in pediatric sepsis.

Recent studies indicated that ALA disrupts the penetration barrier and enhances aminoglycoside antibiotic uptake to eliminate antibiotic-resistant strains (Peng et al. 2015), and takes an important part in cytokine production (Chu et al. 2021). L-alanine supplement could decrease weight and blood glucose in alloxan-induced diabetic rats (Dandare et al. 2021). These results suggest experimental ALA supplement in sepsis might be better for improving sepsis outcome. Florian et al. (Reizine et al. 2022) illustrated that CIT enteral administration could increase ARG availability significantly to restore T cell function and improve sustained immune dysfunction in severe sepsis mice models. In addition, a monocentric, randomized controlled trial that included patients with sepsis or acute respiratory distress syndrome showed that combination of five amino acids (THR, PRO, SER, cysteine and LEU) with enteral feeding increased plasma CIT levels, reduced ALT and alkaline phosphatase levels and improved muscle and gut functionality compared with placebo group (Heming et al. 2022). Therefore, a mechanistic insight of ALA in sepsis is essential in the future.

Except for ALA, our current findings indicate elevated serum THR and LYS levels in patients with sepsis-associated brain injury, which negatively correlate with the *Glasgow* score. THR can cross the blood-brain barrier and protect neuronal cells from zinc’s toxic effects by participating in glutathione (GSH) synthesis and the tricarboxylic acid cycle (Ralph et al. 2010). Moreover, both LYS and THR may inhibit mTOR activity, affecting the early development of human cerebral organoids (Berdenis et al. 2022). Further research is required to elucidate the mechanisms and relationships between LYS or THR and brain injury. Although these results are intriguing, detailed information on the mechanisms by which amino acids contribute to sepsis-associated brain injury remains scarce. Additionally, no amino acids were associated with organ dysfunction in the current population, as evidenced by the lack of correlation between the selected amino acids and the PELOD and PELOD-2 scores. Given the small sample size of the enrolled patients, a larger cohort is crucial for more comprehensive investigations.

Our findings regarding significant alterations in serum amino acid profiles are largely consistent with previous studies in adult patients (Deutz et al. 2021; Druml et al. 2001). With recovery from sepsis, serum levels of GLY, LYS, GLU, PRO, TYR, ASP, CIT, and THR gradually increased, suggesting that amino acids could potentially predict sepsis outcomes. Another study demonstrated that amino acid kinetics could distinguish pediatric severe sepsis from SIRS, and both malnutrition and persistently repressed metabolism are associated with poor outcomes (Spanaki et al. 2018). Prior research has shown that levels of BCAAs are lower in non-survivors of sepsis than in survivors, suggesting a link with mortality (Reisinger et al. 2021). Glutamine can be converted to glutamate by glutamate-dehydrogenase or amino acid transaminase, and glutamate can be converted back to glutamine by glutamine-synthetase. Blaauw et al. (2020) indicated that plasma glutamine levels are associated with ICU patient outcomes, and low glutamine levels correlate with higher APACHE II scores, SOFA scores, and CRP. Given that our study included only two non-survivors, it was not possible to conclusively determine the relationship between serum amino acids and pediatric sepsis outcomes, representing a limitation of our study. To date, there are still limited reports on the value of serum amino acids as effective biomarkers for diagnosis or prognosis in children with sepsis. Exploring the potential values and underlying mechanisms of amino acids in the occurrence, progression, or outcome of pediatric sepsis holds significant promise.

This study presents several limitations. Firstly, it is a single-center study with a small, prospective sample. The reliability and precision of serum amino acids as biomarkers for diagnosing or prognosing sepsis still require validation through larger sample sizes. Secondly, the absence of laboratory data from healthy controls precluded comparisons of significantly altered amino acids with established biomarkers such as CRP, PCT, and IL-6. Thirdly, the concentration and variation of amino acids might be affected by the high incidence of liver injury in septic children. Nonetheless, this study is the inaugural report of serum ALA as a diagnostic biomarker in pediatric sepsis, underscoring the need for further comprehensive clinical and experimental studies.

## Conclusions

Serum amino acid profile is greatly altered in children with sepsis, with serum ALA serving as an optimal biomarker for the early diagnosis of sepsis. Further confirmation of amino acids as biomarkers in sepsis requires a prospective study with a larger sample size.

## Electronic supplementary material

Below is the link to the electronic supplementary material.

Supplementary Material 1

## Data Availability

The datasets used and/or analysed during the current study available from the corresponding author on reasonable request.

## Abbreviations

ARG: Arginine
SBP: Systolic blood pressure
LYS: Lysine
MAP: Mean arterial pressure
HIS: Histidine
CRP: C-reactive protein
ORN: Ornithine
PCT: Procalcitonin
ALA: Alanine
WBC: White blood cell
PRO: Proline
TBIL: Total bilirubin
THR: Threonine
ALT: Alanine aminotransaminase
CIT: Citrulline
AST: Aspartate aminotransferase
GLY: Glycine
γ-GT: γ-glutamyltransferase
SER: Serine
ALB: Albumin
ASN: Asparagine
PT: Prothrombin time
TYR: Tyrosine
INR: International normalized ratio
LEU: Leucine
Fib: Fibrinogen
GLU: Glutamic acid
BUN: Blood urea nitrogen
ILE: Isoleucine
Cr: Creatinine
MET: Methionine
AKI: Acute kidney injury
MET: Methionine
AKI: Acute kidney injury
TRP: Tryptophan
BCAA: Branched chain amino acid
PHE: Phenylalanine
PICU: Pediatric intensive care unit
ASP: Aspartic acid
CI: Confidence interval
VAL: Valine
VIP: Variable importance in projection
MODS: Multiple organ dysfunction syndrome
SIRS: Systemic inflammatory response syndrome
AUC: Area under the receiving operating characteristic curve
UPLC-MS/MS: Ultraperformance liquid chromatography coupled to tandem mass spectrometry
PLS-DA: Partial least squares discriminant analysis

## References

[CR40] Adeva-Andany M, López-Ojén M, Funcasta-Calderón R, Ameneiros-Rodríguez E, Donapetry-García C, Vila-Altesor M, Rodríguez-Seijas J (2014) Comprehensive review on lactate metabolism in human health. Mitochondrion 17:76–100 24929216 10.1016/j.mito.2014.05.007

[CR11] Ahn S, Lee SH, Chung KS, Ku NS, Hyun YM, Chun S, Park MS, Lee SG (2021) Development and validation of a novel sepsis biomarker based on amino acid profiling. Clin Nutr 40(6):3668–3676 34130013 10.1016/j.clnu.2021.05.008

[CR15] Association WM (2013) World medical association declaration of Helsinki: ethical principles for medical research involving. Hum Subj JAMA 310(20):2191–2194 10.1001/jama.2013.281053 24141714

[CR21] Biolo G, Toigo G, Ciocchi B, Situlin R, Iscra F, Gullo A, Guarnieri G (1997) Metabolic response to injury and sepsis: changes in protein metabolism. Nutrition 13(9 Suppl):52S–57S 9290110 10.1016/s0899-9007(97)00206-2

[CR51] Blaauw R, Nel DG, Schleicher GK (2020) Plasma glutamine levels in relation to Intensive Care Unit Patient Outcome. Nutrients 12(2) 10.3390/nu12020402 PMC7071267 32028696

[CR33] Bröer S, Bröer A, Hansen JT, Bubb WA, Balcar VJ, Nasrallah FA, Garner B, Rae C (2007) Alanine metabolism, transport, and cycling in the brain. J Neurochem 102(6):1758–1770 17504263 10.1111/j.1471-4159.2007.04654.x

[CR8] Chen Q, Liang X, Wu T, Jiang J, Jiang Y, Zhang S, Ruan Y, Zhang H, Zhang C, Chen P et al (2022) Integrative analysis of metabolomics and proteomics reveals amino acid metabolism disorder in sepsis. J Transl Med 20(1):123 35287674 10.1186/s12967-022-03320-y PMC8919526

[CR19] Chioléro R, Revelly JP, Tappy L (1997) Energy metabolism in sepsis and injury. Nutrition 13(9 Suppl):45s–51s 9290109 10.1016/s0899-9007(97)00205-0

[CR44] Chu X, Jaeger M, Beumer J, Bakker OB, Aguirre-Gamboa R, Oosting M, Smeekens SP, Moorlag S, Mourits VP, Koeken V et al (2021) Integration of metabolomics, genomics, and immune phenotypes reveals the causal roles of metabolites in disease. Genome Biol 22(1):198 34229738 10.1186/s13059-021-02413-z PMC8259168

[CR35] Coqueiro AY, Raizel R, Bonvini A, Hypólito T, Godois ADM, Pereira JRR, Garcia ABO, Lara RSB, Rogero MM, Tirapegui J (2018) Effects of glutamine and alanine supplementation on central fatigue markers in rats submitted to Resistance Training. Nutrients 10(2) 10.3390/nu10020119 PMC5852695 29370091

[CR36] Dadsetan S, Kukolj E, Bak LK, Sørensen M, Ott P, Vilstrup H, Schousboe A, Keiding S, Waagepetersen HS (2013) Brain alanine formation as an ammonia-scavenging pathway during hyperammonemia: effects of glutamine synthetase inhibition in rats and astrocyte-neuron co-cultures. J Cereb Blood Flow Metab 33(8):1235–1241 23673435 10.1038/jcbfm.2013.73 PMC3734774

[CR38] Dandare SU, Ezeonwumelu IJ, Shinkafi TS, Magaji UF, Adio AA, Ahmad K (2021) L-alanine supplementation improves blood glucose level and biochemical indices in alloxan-induced diabetic rats. J Food Biochem 45(1):e13590 33346923 10.1111/jfbc.13590

[CR13] De Pietri S, Frandsen TL, Christensen M, Grell K, Rathe M, Müller K (2021) Citrulline as a biomarker of bacteraemia during induction treatment for childhood acute lymphoblastic leukaemia. Pediatr Blood Cancer 68(1):e28793 33155402 10.1002/pbc.28793

[CR41] de Souza Galia WB, Biazi GR, Frasson-Uemura IG, Miksza DR, Zaia C, Zaia DAM, de Souza HM, Bertolini GL (2021) Gluconeogenesis is reduced from alanine, lactate and pyruvate, but maintained from glycerol, in liver perfusion of rats with early and late sepsis. Cell Biochem Funct 39(6):754–762 33913177 10.1002/cbf.3637

[CR49] Deutz NEP, Singer P, Wierzchowska-McNew RA, Viana MV, Ben-David IA, Pantet O, Thaden JJ, Ten Have GAM, Engelen M, Berger MM (2021) Comprehensive metabolic amino acid flux analysis in critically ill patients. Clin Nutr 40(5):2876–2897 33946038 10.1016/j.clnu.2021.03.015 PMC8172442

[CR50] Druml W, Heinzel G, Kleinberger G (2001) Amino acid kinetics in patients with sepsis. Am J Clin Nutr 73(5):908–913 11333844 10.1093/ajcn/73.5.908

[CR30] Elia M, Ilic V, Bacon S, Williamson DH, Smith R (1980) Relationship between the basal blood alanine concentration and the removal of an alanine load in various clinical states in man. Clin Sci (Lond) 58(4):301–309 7379455 10.1042/cs0580301

[CR23] Faure M, Choné F, Mettraux C, Godin JP, Béchereau F, Vuichoud J, Papet I, Breuillé D, Obled C (2007) Threonine utilization for synthesis of acute phase proteins, intestinal proteins, and mucins is increased during sepsis in rats. J Nutr 137(7):1802–1807 17585034 10.1093/jn/137.7.1802

[CR1] Fleischmann-Struzek C, Goldfarb DM, Schlattmann P, Schlapbach LJ, Reinhart K, Kissoon N (2018) The global burden of paediatric and neonatal sepsis: a systematic review. Lancet Respir Med 6(3):223–230 29508706 10.1016/S2213-2600(18)30063-8

[CR24] Gale TV, Schieffelin JS, Branco LM, Garry RF, Grant DS (2020) Elevated L-threonine is a biomarker for Lassa fever and Ebola. Virol J 17(1):188 33243278 10.1186/s12985-020-01459-y PMC7690152

[CR14] Goldstein B, Giroir B, Randolph A (2005) International pediatric sepsis consensus conference: definitions for sepsis and organ dysfunction in pediatrics. Pediatr Crit Care Med 6(1):2–8 15636651 10.1097/01.PCC.0000149131.72248.E6

[CR46] Heming N, Carlier R, Prigent H, Mekki A, Jousset C, Lofaso F, Ambrosi X, Bounab R, Maxime V, Mansart A et al (2022) Effect of an enteral amino acid blend on muscle and gut functionality in critically ill patients: a proof-of-concept randomized controlled trial. Crit Care 26(1):358 36397118 10.1186/s13054-022-04232-5 PMC9670468

[CR29] Holeček M (2024) Origin and roles of Alanine and glutamine in Gluconeogenesis in the liver, kidneys, and small intestine under physiological and pathological conditions. Int J Mol Sci 25(13):7037 39000145 10.3390/ijms25137037 PMC11241752

[CR31] Hou Y, Hu S, Li X, He W, Wu G (2020) Amino acid metabolism in the liver: nutritional and physiological significance. Adv Exp Med Biol 1265:21–37 32761568 10.1007/978-3-030-45328-2_2

[CR34] Laustsen C, Lipsø K, Ostergaard JA, Nørregaard R, Flyvbjerg A, Pedersen M, Palm F, Ardenkjær-Larsen JH (2014) Insufficient insulin administration to diabetic rats increases substrate utilization and maintains lactate production in the kidney. Physiol Rep 2(12) 10.14814/phy2.12233 PMC4332212 25501426

[CR25] Liaudet L, Gnaegi A, Rosselet A, Markert M, Boulat O, Perret C, Feihl F (1997) Effect of L-lysine on nitric oxide overproduction in endotoxic shock. Br J Pharmacol 122(4):742–748 9375972 10.1038/sj.bjp.0701419 PMC1564977

[CR10] Mierzchala-Pasierb M, Lipinska-Gediga M, Fleszar MG, Lesnik P, Placzkowska S, Serek P, Wisniewski J, Gamian A, Krzystek-Korpacka M (2020) Altered profiles of serum amino acids in patients with sepsis and septic shock - preliminary findings. Arch Biochem Biophys 691:108508 32712289 10.1016/j.abb.2020.108508

[CR37] Nägeli M, Fasshauer M, Sommerfeld J, Fendel A, Brandi G, Stover JF (2014) Prolonged continuous intravenous infusion of the dipeptide L-alanine- L-glutamine significantly increases plasma glutamine and alanine without elevating brain glutamate in patients with severe traumatic brain injury. Crit Care 18(4):R139 24992948 10.1186/cc13962 PMC4227121

[CR39] Nielsen PM, Mariager C, Mølmer M, Sparding N, Genovese F, Karsdal MA, Nørregaard R, Bertelsen LB, Laustsen C (2020) Hyperpolarized [1-(13) C] alanine production: a novel imaging biomarker of renal fibrosis. Magn Reson Med 84(4):2063–2073 32452096 10.1002/mrm.28326

[CR28] Peng B, Su YB, Li H, Han Y, Guo C, Tian YM, Peng XX (2015) Exogenous alanine and/or glucose plus kanamycin kills antibiotic-resistant bacteria. Cell Metab 21(2):249–262 25651179 10.1016/j.cmet.2015.01.008

[CR42] Peng C, Cheng Y, Ma M, Chen Q, Duan Y, Liu S, Cheng H, Yang H, Huang J, Bu W et al (2024) Mycobacterium tuberculosis suppresses host antimicrobial peptides by dehydrogenating L-alanine. Nat Commun 15(1):4216 38760394 10.1038/s41467-024-48588-4 PMC11101664

[CR32] Pitts RF, Stone WJ (1967) Renal metabolism of alanine. J Clin Invest 46(4):530–538 6021204 10.1172/JCI105554 PMC442036

[CR18] Plummer MP, Deane AM (2016) Dysglycemia and glucose control during Sepsis. Clin Chest Med 37(2):309–319 27229647 10.1016/j.ccm.2016.01.010

[CR17] Pravda J (2014) Metabolic theory of septic shock. World J Crit Care Med 3(2):45–54 24892019 10.5492/wjccm.v3.i2.45 PMC4038812

[CR27] Puskarich MA, McHugh C, Flott TL, Karnovsky A, Jones AE, Stringer KA (2021) Serum levels of branched chain amino acids predict Duration of Cardiovascular Organ failure in septic shock. Shock 56(1):65–72 33156242 10.1097/SHK.0000000000001687 PMC8089113

[CR47] Ralph DM, Robinson SR, Campbell MS, Bishop GM (2010) Histidine, cystine, glutamine, and threonine collectively protect astrocytes from the toxicity of zinc. Free Radic Biol Med 49(4):649–657 20570723 10.1016/j.freeradbiomed.2010.05.023

[CR7] Reisinger AC, Posch F, Hackl G, Marsche G, Sourij H, Bourgeois B, Eller K, Madl T, Eller P (2021) Branched-chain amino acids can predict mortality in ICU Sepsis patients. Nutrients 13(9) 10.3390/nu13093106 PMC8469152 34578983

[CR45] Reizine F, Grégoire M, Lesouhaitier M, Coirier V, Gauthier J, Delaloy C, Dessauge E, Creusat F, Uhel F, Gacouin A et al (2022) Beneficial effects of citrulline enteral administration on sepsis-induced T cell mitochondrial dysfunction. Proc Natl Acad Sci USA 119(8):e2115139119 35173051 10.1073/pnas.2115139119 PMC8872724

[CR20] Sax HC, Hasselgren PO, Talamini MA, Edwards LL, Fischer JE (1988) Amino acid uptake in isolated, perfused liver: effect of trauma and sepsis. J Surg Res 45(1):50–55 3392992 10.1016/0022-4804(88)90020-0

[CR3] Singer M, Deutschman CS, Seymour CW, Shankar-Hari M, Annane D, Bauer M, Bellomo R, Bernard GR, Chiche JD, Coopersmith CM et al (2016) The Third International Consensus definitions for Sepsis and septic shock (Sepsis-3). JAMA 315(8):801–810 26903338 10.1001/jama.2016.0287 PMC4968574

[CR6] Spanaki AM, Tavladaki T, Dimitriou H, Kozlov AV, Duvigneau JC, Meleti E, Weidinger A, Papakonstantinou E, Briassoulis G (2018) Longitudinal Profiles of Metabolism and Bioenergetics Associated with Innate Immune Hormonal inflammatory responses and amino-acid kinetics in severe Sepsis and systemic inflammatory response syndrome in children. JPEN J Parenter Enter Nutr 42(6):1061–1074 10.1002/jpen.1050 29338093

[CR5] Su L, Li H, Xie A, Liu D, Rao W, Lan L, Li X, Li F, Xiao K, Wang H et al (2015) Dynamic changes in amino acid concentration profiles in patients with sepsis. PLoS ONE 10(4):e0121933 25849571 10.1371/journal.pone.0121933 PMC4388841

[CR22] Sun S, Wang D, Dong D, Xu L, Xie M, Wang Y, Ni T, Jiang W, Zhu X, Ning N et al (2023) Altered intestinal microbiome and metabolome correspond to the clinical outcome of sepsis. Crit Care 27(1):127 36978107 10.1186/s13054-023-04412-x PMC10044080

[CR9] Tomé D (2021) Amino acid metabolism and signalling pathways: potential targets in the control of infection and immunity. Nutr Diabetes 11(1):20 34168115 10.1038/s41387-021-00164-1 PMC8223530

[CR16] Träger K, DeBacker D, Radermacher P (2003) Metabolic alterations in sepsis and vasoactive drug-related metabolic effects. Curr Opin Crit Care 9(4):271–278 12883281 10.1097/00075198-200308000-00004

[CR48] van Berdenis A, Kübler R, Hoogeboom JW, Vonk D, Sluijs JA, Pasterkamp RJ, Middeldorp J, Kraneveld AD, Garssen J, Kahn RS et al (2022) Exposure to the amino acids histidine, lysine, and Threonine reduces mTOR activity and affects neurodevelopment in a human cerebral organoid model. Nutrients 14(10) 10.3390/nu14102175 PMC9145399 35631316

[CR4] Vente JP, von Meyenfeldt MF, van Eijk HM, van Berlo CL, Gouma DJ, van der Linden CJ, Soeters PB (1989) Plasma-amino acid profiles in sepsis and stress. Ann Surg 209(1):57–62 2910215 10.1097/00000658-198901000-00009 PMC1493873

[CR2] Wang Y, Sun B, Yue H, Lin X, Li B, Yang X, Shan C, Fan Y, Dong M, Zhang Y et al (2014) An epidemiologic survey of pediatric sepsis in regional hospitals in China. Pediatr Crit Care Med 15(9):814–820 25226498 10.1097/PCC.0000000000000247

[CR12] Weiss SL, Haymond S, Ralay Ranaivo H, Wang D, De Jesus VR, Chace DH, Wainwright MS (2012) Evaluation of asymmetric dimethylarginine, arginine, and carnitine metabolism in pediatric sepsis. Pediatr Crit Care Med 13(4):e210–218 22460770 10.1097/PCC.0b013e318238b5cd PMC3392424

[CR26] Zhang Y, Yu W, Han D, Meng J, Wang H, Cao G (2019) L-lysine ameliorates sepsis-induced acute lung injury in a lipopolysaccharide-induced mouse model. Biomed Pharmacother 118:109307 31404772 10.1016/j.biopha.2019.109307

