# Supplementary material for: Alanine, a potential amino acid biomarker of pediatric sepsis: a pilot study in PICU
Source: Amino Acids. 2024 Jul 27;56(1):48. doi: 10.1007/s00726-024-03408-3 (PMC11281965; doi:10.1007/s00726-024-03408-3)
Supplement: Supplementary file 1 — Supplementary Material 1 [file 726_2024_3408_MOESM1_ESM.docx]

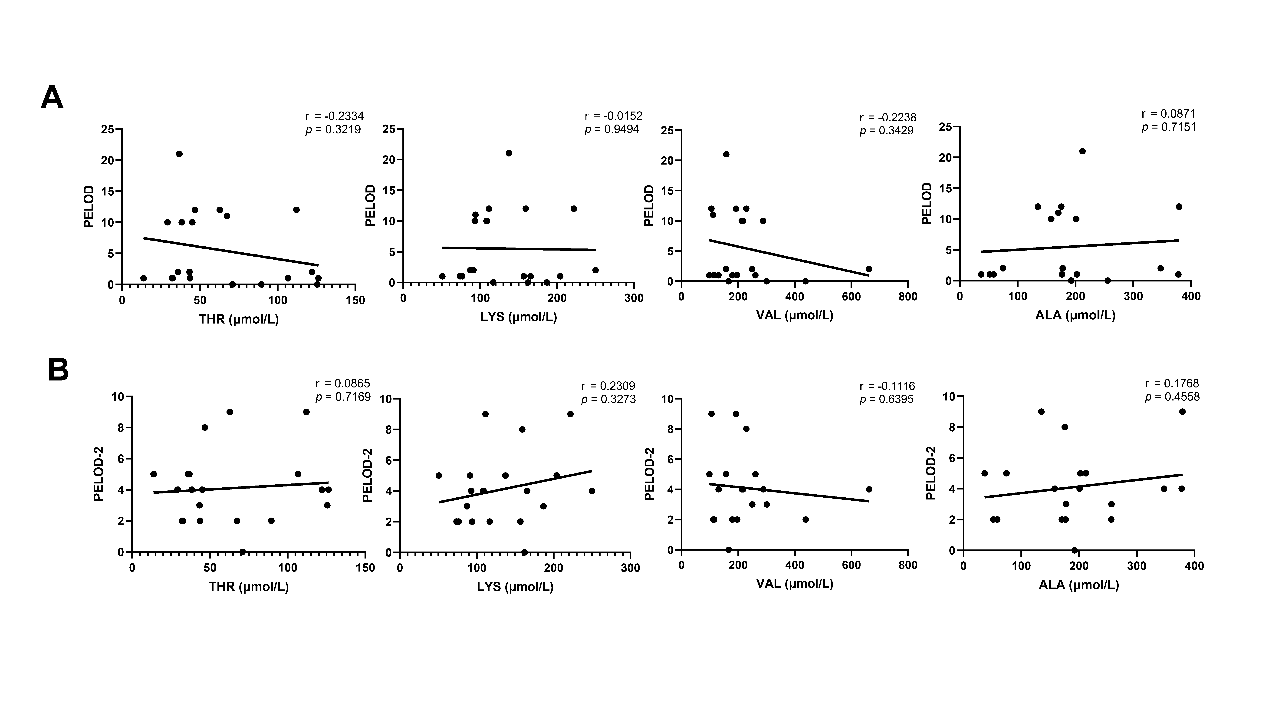


**Supplementary Figure 1. Correlation analysis of serum amino acid levels with the PELOD or PELOD-2 score in septic patients.** **(A).** Correlation between PELOD and serum amino acids (THR, LYS, VAL ALA). **(B).** Correlation between PELOD and serum amino acids (THR, LYS, VAL ALA). Abbreviations: THR: threonine; LYS: lysine; VAL: valine; ALA: alanine. *p* < 0.05 indicated significantly difference.
